# Fixel Based Analysis Reveals Atypical White Matter Micro- and Macrostructure in Adults With Autism Spectrum Disorder: An Investigation of the Role of Biological Sex

**DOI:** 10.3389/fnint.2020.00040

**Published:** 2020-08-13

**Authors:** Melissa Kirkovski, Ian Fuelscher, Christian Hyde, Peter H. Donaldson, Talitha C. Ford, Susan L. Rossell, Paul B. Fitzgerald, Peter G. Enticott

**Affiliations:** ^1^Cognitive Neuroscience Unit, School of Psychology, Deakin University, Geelong, VIC, Australia; ^2^Monash Alfred Psychiatry Research Centre, Monash University, Melbourne, VIC, Australia; ^3^Centre for Human Psychopharmacology, Swinburne University, Melbourne, VIC, Australia; ^4^Centre for Mental Health, Swinburne University, Melbourne, VIC, Australia; ^5^Epworth Centre for Innovation in Mental Health, Epworth Health Care and Central Clinical School Monash University, Melbourne, VIC, Australia

**Keywords:** autism spectrum disorder, fixel based analysis, biological sex, fiber density and cross-section, corpus callosum

## Abstract

Atypical white matter (WM) microstructure is commonly implicated in the neuropathophysiology of autism spectrum disorder (ASD). Fixel based analysis (FBA), at the cutting-edge of diffusion-weighted imaging, can account for crossing WM fibers and can provide indices of both WM micro- and macrostructure. We applied FBA to investigate WM structure between 25 (12 males, 13 females) adults with ASD and 24 (12 males, 12 females) matched controls. As the role of biological sex on the neuropathophysiology of ASD is of increasing interest, this was also explored. There were no significant differences in WM micro- or macrostructure between adults with ASD and matched healthy controls. When data were stratified by sex, females with ASD had reduced fiber density and cross-section (FDC), a combined metric comprised of micro- and macrostructural measures, in the corpus callosum, a finding not detected between the male sub-groups. We conclude that micro- and macrostructural WM aberrations are present in ASD, and may be influenced by biological sex.

## Introduction

Several studies over the last decade have implicated atypical white matter (WM) microstructure in the neurobiology of autism spectrum disorder (ASD; Travers et al., 2012; Aoki et al., 2013; Hoppenbrouwers et al., 2014), a behaviorally characterized, multidimensional neurodevelopmental disorder with varying degrees of symptom severity (American Psychiatric Association, 2013). Previous investigations of WM microstructure in ASD using various diffusion imaging techniques typically show reduced fractional anisotropy (FA) and increased mean diffusivity (MD) in many WM tracts, including the corpus callosum (CC), inferior longitudinal fasciculus (ILF), superior longitudinal fasciculus (SLF), and the arcuate and uncinate fasciculi (AF and UF, respectively). There are, however, inconsistencies regarding these findings (see Travers et al., 2012; Aoki et al., 2013; Hoppenbrouwers et al., 2014 for comprehensive reviews and a meta-analysis), and the nature of WM organization in ASD remains unclear.

Methodological inconsistencies and limitations in diffusion imaging are likely to account for some of the variability observed in the previous literature. Many diffusion tensor imaging (DTI) approaches are unable to reconcile fiber orientation or the presence of multiple fibers within the one voxel (referred to as crossing fibers). These factors have a substantial impact upon approximations, and therefore the interpretability of findings. Advances in the field of diffusion-weighted imaging (DWI) research have resulted in the development of novel approaches that can manage the complexity of such data. Constrained spherical deconvolution (CSD), for example, can estimate fiber orientation at a voxel level (Tournier et al., 2007), and has been suggested to provide more accurate estimates of WM tracts compared to other commonly used DTI techniques (Farquharson et al., 2013). Within the ASD literature, CSD has confirmed WM aberration identified using tract-based spatial statistics (TBSS; Roine et al., 2015), and has revealed WM aberration in tracts linking regions of reduced functional connectivity (McGrath et al., 2013).

Fixel based analysis (FBA) builds on the properties of CSD by accounting for multiple fibers within single voxels, referred to as fixels (Raffelt et al., 2015). Given the tract specific nature of FBA, the metrics used to describe WM structure go beyond the traditional voxel-based metrics of DTI analysis. Rather, FBA provides indices of microscopic fiber density (FD; intra-axonal volume, thought to reflect the density of a population of fibers within voxel), macroscopic fiber cross-section (FC; an estimate of fiber bundle diameter derived from the FD metric), and fiber density and cross-section (FDC; a combined measure of FD and FC; Raffelt D. A. et al., 2017). This technique has been applied to investigate WM in preterm infants (Pannek et al., 2018; Pecheva et al., 2019) and to understand WM development and function in healthy individuals (Genc et al., 2018; Bleker et al., 2019; Mizuguchi et al., 2019), as well as to understand WM aberration in various mental health and medical conditions (Gajamange et al., 2018; Grazioplene et al., 2018; Mito et al., 2018; Mu et al., 2018; Lyon et al., 2019; Dimond et al., 2019; Feshki et al., 2019).

To our knowledge, only one study to date has used FBA to investigate WM aberration in ASD. Dimond et al. (2019) applied FBA to compare WM microstructure between 25 (four females) adolescents/young adults (aged 14–20) with ASD and matched healthy controls (HC; six females). The authors report reduced FD within the CC, the inferior fronto-occipital fasciculus bilaterally, as well as the right AF and UF in the ASD sample compared to HC. Global WM FD was also reported to be reduced in ASD. Importantly, no differences in WM microstructure were observed using a voxel-based approach, highlighting the comparative fidelity of FBA. Furthermore, a negative relationship was identified between social impairment [as measured by the Social Responsiveness Scale–2nd Edition (SRS-2)] and FD within the CC among the ASD group, an association not identified in the HC group, demonstrating the potential clinical relevance of understanding WM structure among this population.

Another factor that may contribute to the inconsistencies seen within the ASD DWI literature, and the ASD literature more broadly, is the failure to consider the role of biological sex on the inherent heterogeneity of ASD, an area of growing interest and importance in the field (Kirkovski et al., 2013; Lai et al., 2015, 2017; Mandy and Lai, 2017). Though limited, the available literature investigating the role of biological sex in ASD does provide evidence to suggest that the structural neuroanatomy (Bloss and Courchesne, 2007; Schumann et al., 2009, 2010; Nordahl et al., 2011; Irimia et al., 2017; Zeestraten et al., 2017) and functional neurobiology (Holt et al., 2014; Alaerts et al., 2016; Kirkovski et al., 2016; Ypma et al., 2016) of ASD differ between males and females with ASD, and beyond expected sexual dimorphisms. Findings from diffusion studies are variable. Work from our group (Kirkovski et al., 2015), as well as others (Beacher et al., 2012), do not indicate sex differences in WM microstructure between those with ASD and HC. Conversely, other studies provide evidence for sex-mediated WM aberration within the frontal (Zeestraten et al., 2017) and temporal (Irimia et al., 2017) tracts in ASD, as well as the CC (Lei et al., 2019). All of these studies, however, used older, voxel-based techniques, and therefore issues associated with multiple and crossing fibers remain unresolved.

In the present study, we sought to replicate and expand upon the findings of Dimond et al. (2019) by using an adult sample and investigating sex differences. First, in line with the findings of Dimond et al. (2019), it was hypothesized that FD will be reduced in adults with ASD compared to matched controls. Regardless of the outcome of this primary analysis, we then sought to explore the effects of biological sex on the WM microstructure among this population. A stratification approach was explicitly adopted to avoid the possible masking of effects of biological sex, as has been previously demonstrated (Holt et al., 2014; Kirkovski et al., 2016; Ecker, 2017), and in line with the previous diffusion MRI studies of sex differences in ASD (Kirkovski et al., 2015; Lei et al., 2019). All analyses were conducted using a whole-brain approach (rather than* a priori* region of interest) to ensure that any relevant sites or tracts could be detected.

## Materials and Methods

### Participants

These data were collected as part of a larger project investigating the role of biological sex in ASD which was approved by the Human Research Ethics Committees of the Alfred Hospital, Monash University, Swinburne University, and Deakin University. The study was conducted following The Code of Ethics of the World Medical Association (Declaration of Helsinki). Participants were recruited *via* flyers placed around university campuses, distributed among various ASD community groups, and posted online (Facebook and Gumtree). Participants were also recruited from the Monash Alfred Psychiatry Research Centre (MAPrc) participant database. All participants provided written informed consent. We have previously published a TBSS paper (Kirkovski et al., 2015) using a mostly overlapping (though not identical) sample from this larger study. Data for 25 individuals with ASD (12 males, 13 females) and 24 age, IQ, and sex-matched HC (12 males, 12 females) are reported in this current study. The Edinburgh Handedness Inventory (Oldfield, 1971) was administered to determine handedness. All HC participants were right-handed. In the ASD group, 16 were right-handed (10 males, six females), four were ambidextrous (one male, three females), and five were left-handed (one male, four female). Participant demographics are presented in Table 1. The Autism Spectrum Quotient (AQ; Baron-Cohen et al., 2001) and Ritvo Autism Asperger Diagnostic Scale-Revised (RADDS-R; Ritvo et al., 2011) were completed by all participants in order summarize traits and characteristics associated with ASD (see Table 2).

**Table 1:** Participant demographics.

		Healthy controls				Autism spectrum disorder					
		Mean (SD)	Mdn	Range	Mean rank	Mean (SD)	Mdn	Range	Mean rank	U	*p*
Age	Total	30.25 (10.08)	27.50	19–56	24.46	30.60 (9.14)	28.00	21–55	25.52	287	0.79
	Male	33.08 (9.44)	30.50	19–49	12.13	33.92 (9.17)	30.50	24–55	12.88	67.50	0.80
	Female	27.42 (10.29)	24.50	19–56	12.67	27.54 (8.29)	23.00	21–44	13.31	74	0.85
Composite IQ^†^	Total	113.25 (13.64)	114.00	82–134	26.63	110.20 (14.60)	111.00	83–139	23.44	261	0.44
	Male	113.50 (15.86)	114.50	82–134	12.71	112.58 (14.89)	115.50	83–31	12.29	69.50	0.89
	Female	113.00 (11.71)	114.00	89–127	14.54	108.00 (14.56)	105.00	89–139	11.58	59.50	0.32

*Note: ^†^Composite IQ measured using the Kaufman Brief Intelligence Test—Second Edition (KBIT-2; Kaufman and Kaufman, 2004)*.

**Table 2:** Summary of self-reported traits and characteristics associated with autism spectrum disorder.

		Healthy controls				Autism spectrum disorder					
		Mean (SD)	Mdn	Range	Mean rank	Mean (SD)	Mdn	Range	Mean rank	U	*p*
AQ
Total	Total	10.83 (5.94)	10.5	2–24	14.04	32.6 (11.09)	34	5–48	35.52	37	<0.001
	Male	11.75 (7.05)	11.5	2–24	6.63	35.75 (7.35)	36	21–48	18.38	1.5	<0.001
	Female	9.92 (4.72)	10	3–18	7.79	29.69 (13.33)	34	5–48	17.81	15.5	<0.001
Social	Total	1.29 (1.52)	1	0–5	13.38	6.84 (2.49)	7	2–10	36.16	21	<0.001
	Male	1.67 (1.72)	1	0–5	6.75	7.5 (1.98)	7	4–10	18.25	3	<0.001
	Female	0.92 (1.24)	0.5	0–4	6.96	6.23 (2.83)	7	2–10	18.58	5.5	<0.001
Switching	Total	2.88 (2.36)	3	0–8	14.90	7.72 (2.51)	8	1–10	34.70	57	<0.001
	Male	3.50 (2.50)	3	0–8	7.67	8.00 (2.13)	8.5	3–10	17.33	14	<0.001
	Female	2.25 (2.14)	2	0–7	7.67	7.46 (2.88)	8	1–10	17.92	14	<0.001
Detail	Total	3.54 (2.55)	3	0–10	17.29	6.72 (2.64)	7	1–10	32.40	115	<0.001
	Male	3.08 (2.39)	3	0–7	7.75	7.17 (2.25)	8	3–10	17.25	15	<0.001
	Female	4.00 (2.73)	3	1–10	10.04	6.31 (2.98)	6	1–10	15.73	42.5	<0.001
Communicate	Total	1.33 (1.74)	0.5	0–6	14.71	6.44 (3.06)	7	0–10	34.88	53	<0.001
	Male	1.92 (2.02)	2	0–6	6.96	7.08 (2.19)	7.5	4–10	18.04	5.5	<0.001
	Female	0.75 (1.22)	0	0–4	7.79	5.85 (3.67)	7	0–10	17.81	15.5	<0.001
Imagine	Total	1.79 (1.44)	1.5	0–5	17.46	4.88 (2.93)	5	0–9	32.24	119	<0.001
	Male	1.58 (1.08)	1.5	0–3	7.00	6.00 (2.49)	6	2–9	18.00	6	<0.001
	Female	2.00 (1.76)	1.5	0–5	10.79	3.85 (3.02)	4	0–8	15.04	51.5	<0.001
RAADS-R											
Total	Total	23.58 (15.25)	24.5	0–64	12.63	132.12 (43.81)	131	49–219	36.88	3	<0.001
	Male	26.83 (18.61)	26.5	0–64	6.50	131.92 (28.07)	129	84–185	18.50	0	<0.001
	Female	20.33 (10.82)	17.5	6–44	6.50	132.31 (55.82)	138	49–219	19.00	0	<0.001
Social relatedness	Total	4.08 (4.13)	3	0–15	12.71	28.76 (14.03)	24	10–59	36.80	5	<0.001
	Male	4.25 (4.41)	3	0–15	6.63	29.42 (14.21)	28	10–59	18.38	1.5	<0.001
	Female	3.92 (4.01)	3	0–12	6.50	28.15 (14.42)	21	13–57	19.00	0	<0.001
Circumscribed interests	Total	10.83 (9.63)	10.5	0–37	12.96	55.28 (18.40)	55	18–84	36.56	11	<0.001
	Male	13.25 (12.13)	13.5	0–37	6.58	57.42 (13.33)	55	36–80	18.42	1	<0.001
	Female	8.42 (5.84)	7.5	0–18	6.58	53.31 (22.47)	57	18–84	18.92	1	<0.001
Sensory motor	Total	6.17 (5.36)	4.5	0–24	13.98	26.6 (13.43)	25	7–50	35.58	35.5	<0.001
	Male	6.25 (6.37)	4	0–24	7.46	23.58 (11.90)	20.5	7–45	17.54	11.5	<0.001
	Female	6.08 (4.42)	5	3–19	7.00	29.38 (14.60)	30	7–50	18.54	6	<0.001
Social anxiety	Total	3.13 (3.22)	3	0–15	13.04	23.20 (7.71)	23	3–34	36.48	13	<0.001
	Male	3.75 (4.18)	3	0–15	6.50	23.00 (4.71)	21.5	17–33	18.50	0	<0.001
	Female	2.50 (1.83)	3	0–5	6.96	23.38 (9.93)	25	3–34	18.58	5.5	<0.001

All participants in the ASD group had received a formal diagnosis (of autistic disorder or Asperger’s disorder) from an external clinician (psychiatrist, psychologist, or pediatrician) before being enrolled in the study, and a copy of the diagnostic report was sighted by the research team. As per the guidelines of the current DSM-5, all participants met the criteria for ASD (American Psychiatric Association, 2013). Prospective participants were not able to take part if they had a history of psychiatric illness (except for mood and anxiety disorders in the ASD group given the prevalence among this population; Matson and Williams, 2014), neurological illness, intellectual disability (IQ < 70), or any contraindications to MRI. Eight participants in this sample self-reported taking one, or a combination, of the following psychoactive medications: selective serotonin reuptake inhibitor (*n* = 6), phenothiazine antipsychotic (*n* = 1), benzodiazepine (*n* = 3), atypical antipsychotics (*n* = 1), norepinephrine reuptake inhibitor (*n* = 1), and serotonin and norepinephrine reuptake inhibitor (*n* = 1).

### Imaging Parameters

Imaging data were collected on a Siemens Tim Trio 3-T MRI scanner with a 32-channel receive-only phased-array head coil (Siemens, Erlangen, Germany). High resolution MPRAGE T1-weighted structural brain images were acquired sagittally using the following parameters; repetition time (TR) = 1,900 ms, echo time (TE) = 2.52 ms, flip angle = 9^o^, acquisition matrix = 256 × 256, inversion time = 900 ms, slice thickness = 1 mm, in-plane resolution = 1 mm isotropic. Dual-Spin echo-planar diffusion weighted images were acquired axially using a 60 direction gradient sequence with the following parameters: *b* value = 2,000 s/mm^2^ with 10 non-diffusion weighted (*b* = 0 s/mm^2^) volumes, flip angle = 90^o^, TR = 9,200 ms, TE = 102 ms, acquisition matrix = 128 × 128, slice thickness = 2 mm, FoV = 256 mm, in-plane resolution = 2 mm isotropic, yielding a total of 46 contiguous slices providing total brain coverage.

### Analysis Protocol

#### Pre-processing

DWI images were processed using pre-processing steps from a recommended FBA pipeline (Raffelt D. A. et al., 2017). Pre-processing included denoising (Veraart et al., 2016), eddy-current correction motion correction (Andersson and Sotiropoulos, 2016), and bias field correction (Tustison et al., 2010). As per the recommended FBA pipeline, images were then up-sampled to a voxel size of 1.3 mm^3^ in all directions using cubic b-spline interpolation (Tournier et al., 2019). This step was included as it can increase anatomical contrast and improve tractography (Dyrby et al., 2014). All preprocessing steps were conducted using commands implemented in MRtrix3 (www.mrtrix.org) or using MRtrix3 scripts that interfaced with external software packages.

For each participant, we estimated response functions for single-fiber WM, gray matter (GM), and cerebrospinal fluid (CSF) using an unsupervised method (Dhollander et al., 2016, 2019). These response functions were then averaged across participants to generate group-level response functions for each tissue type. Single-Shell 3-Tissue CSD (SS3T-CSD) was performed for each participant (using group average response functions) to obtain WM-like fiber orientation distribution (FOD) maps as well as GM-like and CSF-like compartments in all voxels (Dhollander and Connelly, 2016) using MRtrix3Tissue (https://3Tissue.github.io), a fork of MRtrix3 (Tournier et al., 2019). To make the absolute FOD amplitudes comparable between participants, we applied multi-tissue informed intensity normalization in the log-domain (using the *mtnormalise* command in MRtrix3; Raffelt D. et al., 2017; Tournier et al., 2019). Spatial correspondence was achieved by generating a group-specific population template with an iterative registration and averaging approach (Raffelt et al., 2011) using FOD maps from 48 participants (12 females with ASD, 12 males with ASD, 12 HC females, 12 HC males). Each participant’s FOD map was subsequently registered to the population template using FOD-guided non-linear registration (Raffelt et al., 2011, 2012) and segmented to produce a set of discrete fixels (Smith et al., 2013; Raffelt D. A. et al., 2017).

For each participant, we computed FD, FC, and the combined measure of FDC across all WM fixels, as described in Raffelt D. A. et al. (2017). Re-orientation of fixel directions and correspondence of fixels with the template image was performed as previously described (Raffelt D. A. et al., 2017). Finally, we generated a whole-brain tractogram using probabilistic tractography on the population template. To this end, we first generated 20 million streamlines that were subsequently filtered to 2 million streamlines using the spherical-deconvolution informed filtering of tractograms (SIFT; Smith et al., 2013) to reduce reconstruction bias.

### Statistical Analysis

Metrics of apparent FD, FC, and FDC were compared between groups at each WM fixel in the brain using a General Linear Model. Based on two million streamlines from the whole brain tractogram, we performed connectivity-based smoothing and statistical inference using connectivity-based fixel enhancement (Raffelt et al., 2015) using default smoothing parameters (smoothing = 10 mm full-width at half maximum, *C* = 0.5, *E* = 2, *H* = 3). Family-wise error (FWE)-corrected *p*-values were then computed for each fixel using non-parametric permutation testing over 5,000 permutations (Nichols and Holmes, 2002).

In line with our hypotheses, statistical analyses were conducted to compare FD, FC, and FDC between HC and participants with ASD. As per the recommended FBA pipeline, and in line with previous work adopting FBA (e.g., Genc et al., 2018; Pannek et al., 2018), statistical analyses involving FC were conducted using log(FC). Statistical analyses involving log FC or FDC were adjusted for intracranial volume (ICV). ICV did not differ between groups (see Supplementary Table S1 for descriptive statistics). Analyses were repeated with data stratified by sex. A stratification approach was explicitly chosen as the planned approach in line with previous research investigating sex differences in neurobiological mechanisms underlying ASD (Holt et al., 2014; Kirkovski et al., 2016; Ecker, 2017; Lei et al., 2019). Significant fixels (*p* < 0.05, FWE corrected) and fixels demonstrating a trend towards altered white matter structural properties in the ASD group (*p* < 0.10, FWE corrected) were displayed using the mrview tool in MRtrix3. To better appreciate the fiber pathways implicated, we displayed fixels by cropping the whole brain tractogram to streamlines that traversed significant fixels (*p* < 0.05, FWE corrected) or fixels demonstrating a trend towards altered WM structural properties in the ASD group (*p* < 0.10, FWE corrected). Significant streamlines were color-coded by their FWE-corrected *p*-value. All fixel-based statistical analyses and visualizations were performed in MRtrix3.

### Head Motion

To assess in-scanner head motion, we calculated framewise displacement (using the method described in Power et al., 2012) from all volumes (including *b* = 2,000 s and *b* = 0 s) for each participant and then evaluated between group differences on head motion using a 2 (group: ASD vs. HC) by 2 (sex: male vs. female) factorial ANOVA. Results showed a significant main effect for group, *F*_(1,45)_ = 4.28, *p* = 0.044, $\eta_{\text{p}}^{2}$ = 0.09. There was no significant interaction effect, *F*_(1, 45)_ = 0.12, *p* = 0.731, $\eta_{\text{p}}^{2}$ = 0.00, and no significant main effect for sex, *F*_(1, 45)_ = 0.00, *p* = 0.984, $\eta_{\text{p}}^{2}$ = 0.00. Accordingly, head motion was included as a covariate in our analyses (refer to Supplementary Table S2 for descriptive statistics).

## Results

### Comparison of White Matter Properties Between the ASD and Control Groups

**Figure 1:**
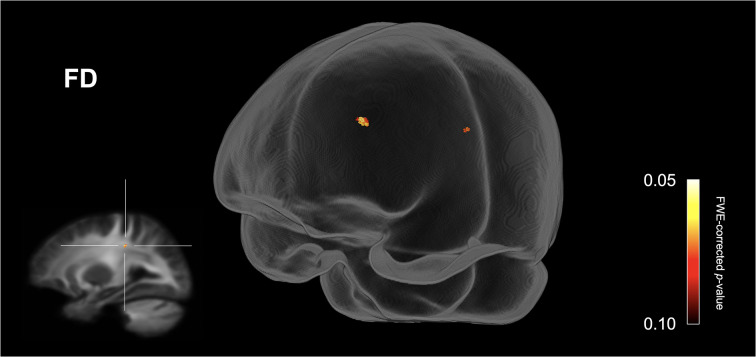
Fiber specific tract reductions in fiber density (FD) in the ASD group (compared to the control group). Streamline segments were cropped from the whole brain tractogram to include only streamlines that traversed fixels showing a trend towards reduced fiber density in the ASD group (*p* < 0.10, FWE corrected). No significant differences were observed at *p* < 0.05, FWE corrected. FWE = Family-wise error.

Figure 1 shows streamline segments associated with fixels demonstrating a trend towards reduced FD (*p* < 0.10, FWE corrected) in the ASD group (relative to the HC group). As can be seen in Figure 1, a trend towards decreased FD in the ASD group was observed within the posterior midbody and/or isthmus of the CC. No areas of increased FD were observed in the ASD group and no significant differences (increases or decreases) were observed for FC or FDC.

### Comparison of White Matter Properties Stratified by Sex

#### Females

**Figure 2:**
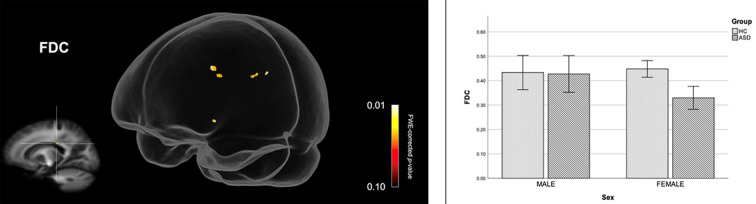
Reduced fiber density and cross-section (FDC) in females with ASD (compared to females in the control group). Streamline segments were cropped from the whole brain tractogram to include only streamlines that traversed fixels showing significantly reduced FDC in females with ASD (*p* < 0.05, FWE corrected). Bar graphs represent mean FDC in each group. Mean FDC was computed for each participant across all fixels showing reduced FDC in females with ASD (*p* < 0.05, FWE corrected). Error bars represent 95% confidence intervals. FWE = family-wise error; HC = healthy control.

**Figure 3:**
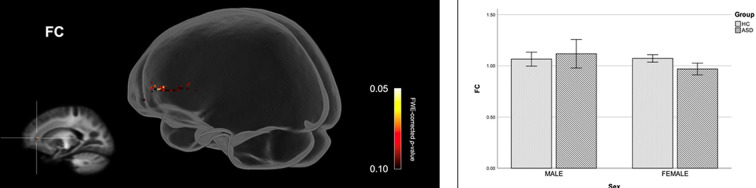
Fiber specific tract reductions in fiber-bundle cross-section (FC) in females with ASD (compared to females in the control group). Streamline segments were cropped from the whole brain tractogram to include only streamlines that traversed fixels showing a trend towards reduced FC in females with ASD (*p* < 0.10, FWE corrected). FWE = Family-wise error. Bar graphs represent mean fiber-bundle cross-section in each group. Mean FC was computed for each participant across all fixels showing a trend towards reduced FC in females with ASD (*p* < 0.10, FWE corrected). Error bars represent 95% confidence intervals. FWE = family-wise error; HC = healthy control.

Figure 2 shows streamline segments associated with fixels demonstrating significantly reduced FDC (*p* < 0.05, FWE corrected) in females with ASD (relative to HC females). We did not observe areas of increased FDC in the ASD group. As can be seen in Figure 2, decreased FDC in females with ASD was found in the posterior midbody and/or isthmus of the CC and the anterior commissure (AC). In females with ASD (relative to HC females), we further observed a trend towards reduced FC (*p* < 0.10, FWE corrected) in the genu of the CC (see Figure 3). No areas of increased FC were observed in the female ASD group and no significant group differences (increases or decreases) were observed for FD.

#### Males

**Figure 4:**
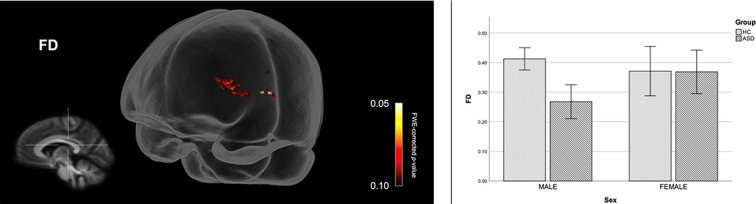
Fiber specific tract reductions in fiber density (FD) in males with ASD (compared to males in the control group). Streamline segments were cropped from the whole brain tractogram to include only streamlines that traversed fixels showing a trend towards reduced FD in males with ASD (*p* < 0.10, FWE corrected). FWE = Family-wise error. Bar graphs represent mean FD in each group. Mean FD was computed for each participant across all fixels showing a trend towards reduced FD in males with ASD (*p* < 0.10, FWE corrected). Error bars represent 95% confidence intervals. FWE = family-wise error; HC = healthy control.

Figure 4 shows streamline segments associated with fixels demonstrating a trend towards reduced FD (*p* < 0.10, FWE corrected) in males with ASD (relative to HC males). As can be seen in Figure 4, a trend towards decreased FD in males with ASD was found around the posterior midbody, isthmus, and splenium of the CC. We did not observe areas of increased FD in the male ASD group and no significant group differences (increases or decreases) were observed for FC or FDC.

## Discussion

This study implemented FBA, a DWI technique capable of accounting for fiber volume and orientation within single voxels and hence addressing important limitations of older DTI approaches, to investigate micro- and macrostructural aberration among adults with ASD. This is the first study to investigate the role of biological sex using this method. Specifically, in line with previous investigations of biological sex and the neuropathophysiology associated with ASD (Holt et al., 2014; Kirkovski et al., 2015, 2016, 2018; Lei et al., 2019), data were stratified by sex and indices of WM micro- and macrostructure were compared within sex. For data such as that which is presented in the present study, this approach has been shown to reveal effects that might be masked using statistical models that do not adopt a stratification approach. While not statistically significant, there was a trend indicative of reduced FD within the CC, at the posterior midbody/isthmus, in the ASD group compared to age, sex, and IQ matched HC participants. This was replicated between males, but not females when the data were stratified by sex; again, however, this result did not reach statistical significance. Finally, comparing the female sub-samples, FDC at the CC (posterior midbody/isthmus) was significantly reduced for females with ASD compared to their age and IQ matched female counterparts. There was also a non-significant trend level difference to suggest reduced FC in the genu of the CC for the female ASD group compared to matched HC participants. The reverse contrasts did not reveal any areas showing increased FD, FC, or FDC in the ASD group when comparing the whole sample, nor when data were stratified by sex. A vital point when considering these results is that our overall sample size is relatively comparable to that presented in the only other published FBA investigation of ASD (Dimond et al., 2019), and therefore the subtlety of our results, particularly concerning our trend level findings when comparing the entire sample, might reflect upon, and inform, two pertinent avenues of ASD research.

Firstly, as expected, we present evidence in support of the notion that the pattern of neuropathophysiology observed among those with ASD differs between males and females when compared to matched counterparts. Our sample comprised substantially more females than that of Dimond et al. (2019), and therefore we must consider the extent to which this might affect the results observed between the studies. Here, the only observable group difference to reach statistical significance was between the female sub-groups, which might indicate greater neuropathophysiology among females with ASD. To date, research investigating sex differences in WM microstructure in ASD is scarce and inconsistent (Beacher et al., 2012; Kirkovski et al., 2015; Irimia et al., 2017; Zeestraten et al., 2017; Lei et al., 2019). Our findings, however, do align with that of Lei et al. (2019), who report no significant group differences between ASD and control groups when males are females were included, nor any difference between the male-only subsamples. Interestingly, the authors report reduced FA in females with ASD at the cingulate, inferior fronto-occipital fasciculus, ILF, SLF, UF, anterior thalamic radiation, corticospinal tract, forceps major, and the forceps minor (Lei et al., 2019). Concerning sex differences in the neurobiology of ASD more broadly, it was previously considered that females with ASD may have more severe and widespread neuropathophysiology, despite seemingly less severe behavioral profiles (Kirkovski et al., 2013). Indeed, this is supported by early volumetric studies of ASD (Bloss and Courchesne, 2007; Schumann et al., 2009, 2010). Furthermore, in considering our finding of reduced FDC in females with ASD compared to their unaffected counterparts, we must consider this theory as well as the FDC metric itself. As described above, FDC combines measures of WM micro- (FD) and macro- (FC) structure, neither of which alone revealed significant differences between our female subgroups. This approach, which is arguably more sensitive than FD or FC alone, indicates total intra-axonal volume changes (Raffelt D. A. et al., 2017; Mito et al., 2018). Further corroborating the aforementioned theory of greater neurobiological aberration among affected females, the lack of difference on our FDC measure between the male sub-groups might indicate that the main source of WM abnormality among this sub-sample was micro-structural, in line with the observed trends, and therefore combining this with FC, a macrostructural measure, to provide the FDC metric did not yield any significant differences between groups.

The findings presented here, along with those of Lei et al. (2019), highlight the benefit of sex-stratification when investigating sex differences in ASD. Such an approach has been taken in several studies investigating sex differences in the neuropathophysiology of ASD (Holt et al., 2014; Kirkovski et al., 2015, 2016, 2018; Lei et al., 2019), the majority of which identified sex-related differences that were masked within whole group analyses. Ecker, 2017 discussed the importance of stratification approaches in such research, highlighting that not only would such an approach be beneficial in reducing confounds associated with the phenotypic heterogeneity of ASD, but also the common situation where included males far outnumber included females.

Second, while inconsistent with our hypotheses, the results presented here are, for the most part, in line with the findings of our previous study investigating WM pathology in ASD (Kirkovski et al., 2015). In our earlier study, we found no group differences in DTI based measures of WM microstructure. The results presented in this study, as well as our previous work with a largely overlapping sample, might be reflective of some degree of age-related “normalization” of the neuropathophysiology observed in ASD, a phenomenon identified in brain volume research in this population over a decade ago (Courchesne, 2004; Courchesne et al., 2011; Redcay and Courchesne, 2005). Indeed, more recent reviews highlight the implications of age on research investigating brain volume and morphometry, structural integrity, and function among ASD populations (Duerden et al., 2012; Pua et al., 2017). Of particular relevance to this study, there is evidence in the ASD literature to indicate a relationship between age and tensor-based measures of WM microstructure (FA, MD, axial and radial diffusivity), highlighting the importance of age when interpreting such data (Bakhtiari et al., 2012; Kleinhans et al., 2012; Karahanoğlu et al., 2018). Karahanoğlu et al. (2018) report a switch in the directionality of this relationship, whereby in younger participants (late childhood/adolescence) FA and axial diffusivity were higher in the ASD compared to control group, while among the older sub-sample (young adulthood) the opposite pattern was observed. When splitting their samples by age, two separate studies (Ameis et al., 2011; Bakhtiari et al., 2012) report no differences between the older samples. Importantly, both samples were younger than those presented here. There are, however, many reports of altered WM structure among adults with ASD (see Travers et al., 2012; Aoki et al., 2013; Hoppenbrouwers et al., 2014), including the present study. Indeed, we see an overlap between the areas implicated in our study and that of Lei et al. (2019). One important difference between these two studies is the age of participants, with the sample presented by Lei et al. (2019) comprising children, adolescents, and young adults (4–21 years), while the present study comprises only adults. In considering the effects of age on WM structure, as described above, the widespread aberration observed in the younger sample “may normalize” to some extent in older samples, whereby only areas that were perhaps more aberrant remain detectable among older samples.

Concerning the role of the CC and the pattern of observed results within this study, the CC is one of the most heavily implicated brain regions in research using various diffusion protocols to investigate WM in ASD (Travers et al., 2012; Hoppenbrouwers et al., 2014), and was a tract identified as showing reduced FD among ASD participants in the only other FBA comparison of individuals with and without ASD by Dimond et al. (2019). The CC is typically described in terms of five sections, including, from anterior to posterior, the rostrum, genu, body, isthmus, and splenium, to better characterize locations along the tract. Here, the areas of the CC most indictive of having WM aberration included the body and isthmus, located between the body and splenium, with some indication of the genu being affected among the female ASD subsample. Our findings are in line with those reported by Sui et al. (2018), who, using diffusion kurtosis imaging (DKI), demonstrated that individuals with ASD showed reduced *f*_axon_, a DKI metric indicative of intra-axonal water, in segment 3, 4, and 5 of the CC, corresponding with the midbody and isthmus, as well as the splenium. Further, in their meta-analysis of CC area, Frazier and Hardan, 2009 report that the greatest reduction of CC size in ASD can be observed in the midbody. A recent post-mortem investigation of nine ASD brains demonstrated overall reductions in the total number of axons across all parcellations of the CC (Wegiel et al., 2018), again supporting the use of FBA for investigating WM in ASD.

Given that the CC is the largest commissural WM bundle connecting the left and right hemispheres of the brain, the interhemispheric signal transfer is likely to be compromised in ASD (Valenti et al., 2020). Another consideration in terms of the implications of these findings is the functional relevance of the fiber projections stemming from these areas. It is well established that the posterior midbody projects to somatosensory regions, while the isthmus includes temporal and parietal projections (de Lacoste et al., 1985; Hofer and Frahm, 2006). Concerning the genu, also implicated by Dimond et al. (2019), fibers within this section of the CC cross through the forceps minor, a region implicated by Lei et al. (2019), and one that has projections to the frontal cortex. Therefore, the CC segments implicated in the present study project WM fibers to regions involved in the two core domains of autistic symptomatology; namely, social communication and repetitive behaviors/restricted interests (which, under DSM-5, now includes sensory sensitivities (American Psychiatric Association, 2013). Regarding the former, temporoparietal regions are strongly implicated in the cognitive processes involved in social understanding (Patriquin et al., 2016), while sensory difficulties experienced by affected individuals have been attributed to functional deficits within the somatosensory cortex (Lajiness-O’Neill et al., 2014). Indeed, Kana et al. (2014) address the potential relationship between structural and functional neuropathophysiology in ASD by combining task-based fMRI and DTI to investigate the structural and functional neural correlates of theory of mind (ToM), a cognitive model of social understanding, in ASD. As expected, blood oxygen level-dependent (BOLD) response was reduced at the temporoparietal junction during ToM in the ASD group, and FA in the WM underlying this region was also reduced.

In further considering the potential role and influence of biological sex on the structural and functional neuropathophysiology in ASD, two functional magnetic resonance imaging (fMRI) studies exploring sex differences in social cognition revealed the expected pattern of atypical neurobiological processes among males with ASD, but not females (Holt et al., 2014; Kirkovski et al., 2016). There are two important factors to consider here. First, while the aforementioned effects at a functional level may be attributed to behavioral or learning processes, the extent to which structural abnormalities, such as those presented in this paper, are implicated must be considered. Concerning the fMRI studies described above, along with the knowledge that isthmus projections reach temporal and parietal areas, we demonstrate a plausible link between structural and functional neuropathophysiological sex differences in ASD that warrants further investigation.

Despite using a cutting-edge approach to investigate a largely overlooked area of ASD (i.e., the role of biological sex), the present study is not without limitation. Though our sample was well matched for age, sex, and IQ, all enabling stratification of data and investigation of the role of biological sex between those with and without ASD, our stratified samples are small, hence reducing statistical power. An important consideration, too, is that there are limitations regarding the interpretations that can be made based on such analytical approaches (Nieuwenhuis et al., 2011). While we do not discount the value of testing such data for interaction effects, in considering our small sample, as well as the effects observed in similar research in this area (Holt et al., 2014; Kirkovski et al., 2015, 2016, 2018; Ecker, 2017; Lei et al., 2019), we assert that our approach has merit. Further, while some of our samples reported taking psychotropic medication, given our small sample size we were not able to account for this in the analysis (although Dimond et al., 2019 report that medication did not have any substantial impacts on their findings). Therefore, these findings should be considered preliminary, and we encourage future studies of ASD to also consider the role of biological sex. It is also important to acknowledge that many factors beyond biological sex, and the scope of this paper, contribute to the heterogeneous nature of ASD, and should also be considered.

In conclusion, we applied FBA to investigate WM micro- and macrostructure among adults with ASD, without intellectual disability, and also explored the influence of biological sex. We provide evidence for micro-and macrostructural aberration among adult females with ASD, but not for males. Our findings on the role of biological sex are of great interest and importance to the ASD literature. While the pattern of results was convergent to some extent, we show that males and females with ASD differ in terms of the strength of the aberration observed and the underlying structural properties. In considering that the vast majority of research in ASD is heavily biased towards the male ASD population, the ramifications of the historical approach of researchers excluding (actively or otherwise) affected females can be detrimental to our understanding of the condition, and therefore our ability to accurately and appropriately identify, understand, and treat those affected. It is imperative that future research further investigates sex differences in the neurobiological basis of ASD, and we strongly encourage such research to adopt a stratification approach.

## Data Availability Statement

The datasets generated for this study are available on request to the corresponding author.

## Ethics Statement

The studies involving human participants were reviewed and approved by Alfred Human Research Ethics Committee, The Alfred, Melbourne, VIC, Australia. The patients/participants provided their written informed consent to participate in this study.

## Author Contributions

MK contributed to the study design, data acquisition and analysis, interpretation of results, and preparation of the manuscript. IF and CH contributed to data analysis, interpretation of the results and preparation of the manuscript. PD and TF contributed to the interpretation of the results, and preparation of the manuscript. SR contributed to the study design and data acquisition. PF and PE oversaw the entire study, including conceptualization, analysis, and manuscript preparation. All authors contributed to the article and approved the submitted version.

## Conflict of Interest

PF has received equipment for research from MagVenture A/S, Medtronic Limited, Neuronetics and Brainsway Limited and funding for research from Neuronetics. He is on scientific advisory boards for Bionomics Limited and LivaNova and is a founder of TMS Clinics Australia. The remaining authors declare that the research was conducted in the absence of any commercial or financial relationships that could be construed as a potential conflict of interest.
