# Supplementary material for: Fixel Based Analysis Reveals Atypical White Matter Micro- and Macrostructure in Adults With Autism Spectrum Disorder: An Investigation of the Role of Biological Sex
Source: Front Integr Neurosci. 2020 Aug 13;14:40. doi: 10.3389/fnint.2020.00040 (PMC7438780; doi:10.3389/fnint.2020.00040)
Supplement: Supplementary file 1 [file Table_1.docx]

Supplementary Material

Fixel based analysis reveals atypical white matter micro- and macrostructure in adults with autism spectrum disorder: An investigation of the role of biological sex

Supplementary Table S1. Descriptive statistics for intracranial volume (ICV)

|  |  | Healthy Controls | | | Autism Spectrum Disorder | | |
| --- | --- | --- | --- | --- | --- | --- | --- |
|  |  | Mean (SD) | Range | Mdn | Mean (SD) | Range | Mdn |
| ICV | Total | 1.5 (0.14) | 1.23 – 1.74 | 1.50 | 1.52 (0.17) | 1.28 – 2.30 | 1.50 |
| (litres) | Male | 1.54 (0.16) | 1.23 – 1.74 | 1.54 | 1.60 (0.18) | 1.35 – 2.30 | 1.59 |
|  | Female | 1.46 (0.10) | 1.30 – 1.62 | 1.46 | 1.44 (0.11) | 1.28 – 1.63 | 1.42 |

Supplementary Table S2. Descriptive statistics for frame wise displacement (FWD)

|  |  | Healthy Controls | | | Autism Spectrum Disorder | | |
| --- | --- | --- | --- | --- | --- | --- | --- |
|  |  | Mean (SD) | Range | Mdn | Mean (SD) | Range | Mdn |
| **Pre-correction** | |  |  |  |  |  |  |
| FWD mm (including b=2000s and b=0s) | Total | 0.99 (0.26) | 0.64 - 1.62 | 0.94 | 1.23 (0.64) | 0.61 - 3.87 | 1.24 |
|  | Male | 0.97 (0.24) | 0.64 - 1.37 | 0.94 | 1.32 (0.33) | 0.68 - 1.74 | 1.30 |
|  | Female | 1.01 (0.30) | 0.67 - 1.62 | 0.96 | 1.26 (0.85) | 0.61 - 3.87 | 1.09 |
| FWD mm (including b=2000s only) | Total | 0.61 (0.15) | 0.42 - 0.89 | 0.56 | 0.78 (0.51) | 0.41 - 3.13 | 0.71 |
|  | Male | 0.52 (0.1) | 0.42 - 0.79 | 0.50 | 0.69 (0.17) | 0.42 - 0.99 | 0.66 |
|  | Female | 0.69 (0.14) | 0.44 - 0.89 | 0.70 | 0.86 (0.7) | 0.41 - 3.13 | 0.75 |
| **Post-correction** | |  |  |  |  |  |  |
| FWD mm (including b=2000s and b=0s) | Total | 0.83 (0.22) | 0.48 - 1.22 | 0.85 | 0.98 (0.32) | 0.54 - 1.8 | 0.92 |
|  | Male | 0.75 (0.2) | 0.48 - 1.06 | 0.79 | 1.01 (0.4) | 0.54 - 1.8 | 0.90 |
|  | Female | 0.91 (0.22) | 0.5 - 1.22 | 0.96 | 0.96 (0.23) | 0.68 - 1.32 | 0.92 |
| FWD mm (including b=2000s only) | Total | 0.55 (0.09) | 0.38 - 0.75 | 0.54 | 0.59 (0.13) | 0.43 - 1.08 | 0.56 |
|  | Male | 0.53 (0.09) | 0.38 - 0.66 | 0.54 | 0.55 (0.1) | 0.43 - 0.75 | 0.54 |
|  | Female | 0.57 (0.09) | 0.42 - 0.75 | 0.56 | 0.63 (0.15) | 0.51 - 1.08 | 0.59 |

Note. In light of evidence that motion estimates calculated from diffusion data may be influenced by differences in contrasts (Ben‐Amitay et al., 2012), we present motion estimates calculated from the entire sequence as reported in the main manuscript, and also for b=2000s only.

References

Ben‐Amitay, S., Jones, D. K., & Assaf, Y. (2012). Motion correction and registration of high b‐value diffusion weighted images. *Magnetic resonance in medicine*, *67*(6), 1694-1702.
